# Peptides in wound healing: A comprehensive review of their roles, challenges, and hydrogel-based delivery systems

**DOI:** 10.17179/excli2025-8778

**Published:** 2025-12-01

**Authors:** Rafl M. Kamil, Shaik Nyamathulla, Syed Mahmood

**Affiliations:** 1 Department of Pharmaceutical Technology, Faculty of Pharmacy, Universiti Malaya, 50603 Kuala Lumpur, Malaysia; 2 Faculty of Medicine, Universiti Malaya Research Centre for Biopharmaceuticals and Advanced Therapeutics (UBAT), Universiti Malaya, 50603 Kuala Lumpur, Malaysia; 3 Centre of Advanced Materials (CAM), Faculty of Engineering, Universiti Malaya, 50603, Kuala Lumpur, Malaysia; 4 Faculty of Pharmaceutical Sciences, Chulalongkorn University, Pathum Wan, Bangkok, 10330, Thailand

**Keywords:** peptides, wound healing, biomaterials, hydrogels, tissue regeneration

## Abstract

Chronic wounds are characterized by prolonged healing durations and disrupted progression through the normal phases of wound healing, hemostasis, inflammation, proliferation, re-epithelialization and remodeling. These wounds are often complicated by persistent infections and underlying conditions like diabetic mellitus, which hinders effective tissue regeneration. Traditional dressings provide limited therapeutic benefits; therefore, recent advancements in wound care have introduced peptide-based therapies that have gained considerable attention for their multifunctional roles in modulating wound repair. Peptides possess intrinsic antimicrobial, anti-inflammatory, angiogenic, and pro-regenerative properties, enabling them to regulate diverse cellular and molecular events across all stages of healing. This review highlights the mechanistic roles of therapeutic peptides in regulating and orchestrating wound healing applications. We further classify bioactive peptides derived from microbial, animal, and plant sources with documented roles in wound healing, and also address synthetic peptides engineered for wound healing. We discussed the peptide-based hydrogels, recent advancements in peptide-based hydrogels in wound healing, and also those hydrogels that are currently under investigation in clinical trials. The primary objective of this review is to provide the readers a detailed overview of the advancements in wound healing studies especially peptide incorporated hydrogels.

See also the graphical abstract(Fig. 1).

## Introduction

The skin is the largest organ in the human body, accounting for approximately 16 % of the total body weight. It serves as the outermost barrier against environmental, microbial, and physical insults. The skin is composed of three main layers; epidermis, dermis, and hypodermis (also known as the subcutaneous layer). Each layer contributes to structural integrity, homeostasis, and immune defense (McKnight et al., 2022[87]; Wong et al., 2016[149]). When this barrier is compromised by injury, burns, trauma, surgery, or pathological conditions such as diabetes or peripheral vascular disease, the body initiates a complex biological process known as wound healing. This process aims to restore tissue integrity, reduce microbial load, and re-establish homeostasis through a tightly regulated cascade of cellular and molecular events (Sorg et al., 2017[132]). 

**Figure 2:**
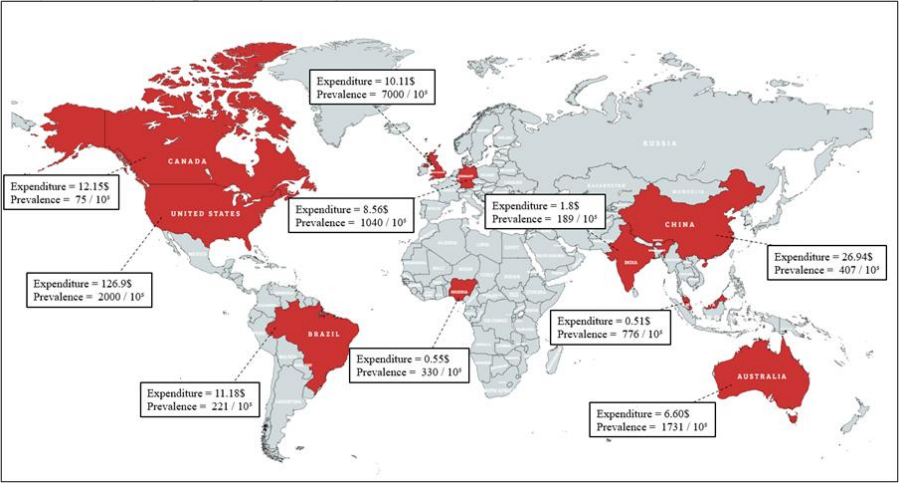
The illustration shows the total healthcare expenditure on wound care (in billion USD per year) and the prevalence of wounds (per 100,000 population) across selected countries (Ref: Wounds Australia, 2024; Queen and Harding, 2024; Ruiz and Lima, 2022; Queen and Botros, 2024; Hopkins et al., 2015; Queen and Harding, 2023; Jiang et al., 2020; Healthcare-in-Europe, 2008; Heyer et al., 2016; Morat and Ajemi, 2024; Iyun et al., 2024; Kharrati, 2024; Sharma et al., 2024; Sen, 2021).

The earliest recorded wound treatments date back to ancient Egyptian script, Ebers Papyrus, they used honey and natural substances with bandages for their antimicrobial and absorbent properties to treat wounded individuals. In ancient Greece and Rome, physicians such as Galen emphasized moisture maintenance for optimal healing. Significant progress occurred in the 19^th^ century, when Joseph Lister, guided by Pasteur's germ theory, introduced antiseptic practices using phenol, which drastically reducing surgical infections and revolutionized wound care (Ahmad et al., 2020[6]; Michaleas et al., 2022[90]). Today, chronic wounds significantly burden healthcare systems due to prolonged healing time and high treatment costs (Powers et al., 2016[109]). As shown in Figure 2(Fig. 2) (Reference in Figure 2: Wounds Australia, 2024[150]; Queen and Harding, 2024[112]; Ruiz and Lima, 2022[121]; Queen and Botros, 2024[111]; Hopkins et al., 2015[54]; Queen and Harding, 2023[113]; Jiang et al., 2020[63]; Healthcare-in-Europe, 2008[51]; Heyer et al., 2016[53]; Morat and Ajemi, 2024[94]; Iyun et al., 2024[61]; Kharrati, 2024[71]; Sharma et al., 2024[125]; Sen, 2021[122]), the United States spends approximately $126.95 billion annually on chronic wound care, followed by substantial expenditures in Germany, Canada, China, Australia, and other countries.

Given these burdens and the urgent demand for more effective interventions peptides have attracted a significant attention for their antimicrobial, immunomodulatory, and regenerative properties. Peptides like LL-37 and β-defensins promote fibroblast and keratinocyte activity, reduce inflammation, and prevent infection (Ahmad et al., 2024[5]). Because of their size and multifunctional activity, peptides are promising agents for chronic wound treatment, as they exert their benefits through multiple pathways that are dysregulated in chronic wounds. Some of these pathways include promoting re-epithelialization by stimulating keratinocyte and fibroblast migration and proliferation, improving angiogenesis by upregulating vascular endothelial growth factor (VEGF), and accelerate extracellular matrix (ECM) remodeling by modulating the activity of matrix metalloproteinases (MMPs) and tissue inhibitors of metalloproteinases (TIMPs) (Gomes et al., 2017[44]; Kumar et al., 2024[74]). Many peptides also possess an immunomodulatory properties that shift macrophage phenotypes from a pro-inflammatory (M1) to a regenerative (M2) state, thereby resolving persistent inflammation commonly seen in chronic wounds (Nazari et al., 2025[99]). Their high specificity, low immunogenicity, and potential for combinatorial modification make peptides highly versatile candidates for wound therapy (Guan et al., 2022[46]). However, peptide instability remains a major limitation, which prompt their integration of peptides into delivery platforms such as hydrogels. These systems enhance peptide retention at the wound site, protect against enzymatic degradation, and sustain therapeutic activity, eventually improving healing outcomes (Kumar et al., 2024[74]).

This review highlights the mechanistic roles of therapeutic peptides in orchestrating wound healing, and briefly explores their integration into advanced delivery systems such as hydrogels to improve stability and bioavailability. By precisely targeting specific biological pathways, peptide-based interventions represent a promising frontier in chronic wound management. We further presented bioactive peptides derived from microbial, animal, and plant sources with documented roles in wound healing, and discussed synthetic peptides engineered for wound healing applications. We also discussed the commercial aspects of peptide-based hydrogels used in wound care, recent advancements in their application to wound healing, and their current clinical trials. Furthermore, this review also aims to provide a comprehensive guide for professionals and researchers from diverse scientific communities, including pharmaceutical sciences, clinical sciences, biomaterials, and individuals engaged in wound healing-related studies.

## Classification of Wounds

The classification of wounds can be made based on various parameters. An accurate classification is essential for diagnosing the severity of the wound, predicting its healing pace, and selecting the most effective treatment approach (Abazari et al., 2022[1]). Thereby, Table 1(Tab. 1) (References in Table 1: Abazari et al., 2022[1]; Boersema et al., 2021[13]; Percival, 2002[104]; Rodrigues et al., 2019[120]; Stefanou et al., 2020[134]) summarizes the key classification criteria for wounds, which are as follows: nature, etiology, depth, level of pollution, and healing process time.

## Peptides’ Roles in Orchestrating Wound Healing Phases

**Figure 3:**
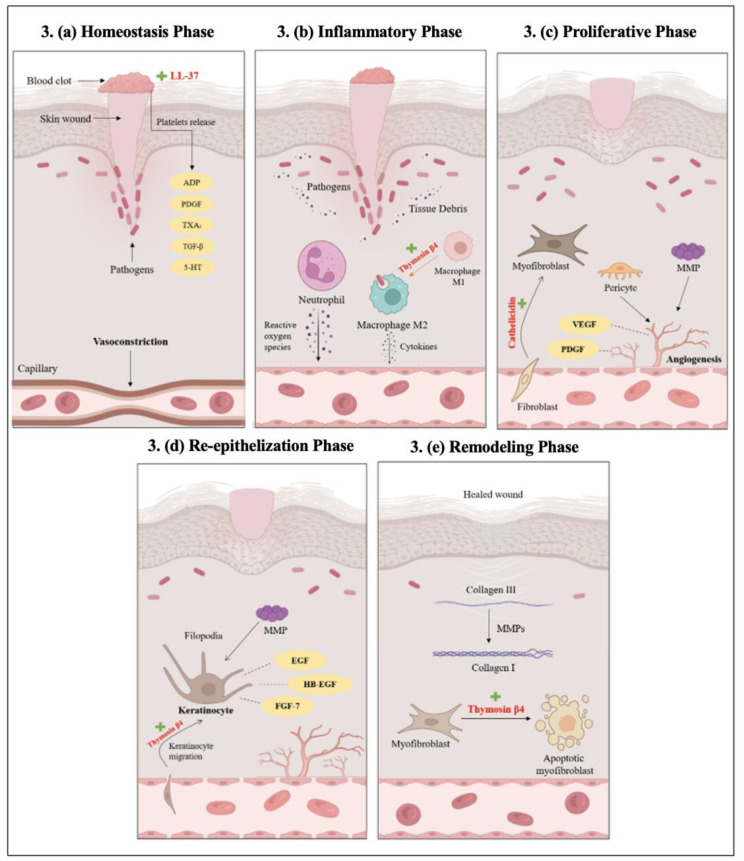
Schematic representation of the five sequential phases of wound healing and the associated roles of key peptides where (a) Hemostasis phase that is characterized by platelet aggregation and vasoconstriction mediated by secreted molecules including ADP, PDGF, TXA_2_, TGF-β, and 5-HT; Here in peptides such as LL-37 can induce further clot formation. (b) Inflammatory phase were neutrophils and macrophages clear pathogens. Peptides such as Thymosin β4 can facilitate macrophage transition. (c) Proliferative phase were fibroblasts, myofibroblasts, and pericytes coordinate ECM deposition and angiogenesis. Peptides such as Cathelicidin can support this phase. (d) Re-epithelialization phase in which keratinocyte migration and proliferation occur, and it's driven by MMPs and growth factors (EGF, HB-EGF, FGF-7), this can be enhanced by peptides such LL-37 and Thymosin β4. Lastly, (e) Remodeling phase where collagen maturation and tissue contraction take place, and it's regulated by MMPs and myofibroblast apoptosis which can be facilitate by peptides such as Thymosin β4. (VEGF, Vascular Endothelial Growth Factor; TGF-β, Transforming Growth Factor-beta; MMP, Matrix Metalloproteinase; EGFR, Epidermal Growth Factor Receptor).

Wound healing is a complex, multistep process characterized by a cascade of biological events that can be divided into five overlapping but distinct phases: hemostasis, inflammation, proliferation, re-epithelialization, and remodeling (Rodrigues et al., 2019[120]). Immediately after injury, the hemostasis phase begins to prevent blood loss through rapid vasoconstriction and platelet aggregation (Golebiewska and Poole, 2015[43]). Activated platelets release a variety of vasoactive substances and growth factors including ADP, TXA2, PDGF, TGF-β, and serotonin (5-HT), which facilitate clot formation and recruit inflammatory cells (Beura et al., 2022[12]). Peptides such as LL-37 can further support the process of clot formation (Duan et al., 2022[29]) as illustrated in Figure 3a(Fig. 3).

As the hemostatic plug stabilizes, the inflammatory phase begins with neutrophil infiltration, leading to the release of reactive oxygen species, proteases, and antimicrobial peptides such as LL-37 (Adnan et al., 2025[3]). LL-37 exerts direct antimicrobial effects and modulate immune responses by neutralizing endotoxins, recruiting immune cells, and enhancing cytokine production (Agier et al., 2015[4]). Under normal inflammatory phase of wound healing, the monocytes differentiate from M1 to M2 macrophage phenotypes. This transition is essential for tissue debridement, secretion of tissue repair cytokines and promote wound healing. This process can be further facilitated by peptides such as Thymosin β4, which activates macrophages, thereby promoting infection control and resolution of inflammation (Yu et al., 2025[162]) as shown in Figure 3b(Fig. 3).

The proliferative phase then reconstructs the wound tissue through fibroblast proliferation, ECM synthesis, and angiogenesis. Endothelial cells and pericytes form new capillaries under the influence of VEGF and PDGF, while fibroblasts deposit collagen and glycoproteins (Rodrigues et al., 2019[120]). Thymosin-β4 significantly enhances this phase by stimulating fibroblast migration, upregulating VEGF, promoting neovascularization, and exerting cytoprotective effects against oxidative stress (Srinivasan et al., 2025[133]). As granulation tissue forms, keratinocytes at the wound edges initiate re-epithelialization by detaching from their basement membrane, extending filopodia, and migrating across the wound bed. This process is guided by growth factors such as EGF, HB-EGF, and FGF-7, and is further accelerated by peptides such as LL-37, which activates EGFR signaling to promote keratinocyte migration and proliferation (Tomic-Canic et al., 2018[139]; Wang et al., 2016[144]). Thymosin β4 also enhances this phase by supporting keratinocyte motility, ECM remodeling, and cytoskeletal organization, thereby facilitating rapid epithelial coverage (Yu et al., 2025[162]) as illustrated in Figure 3c and 3d(Fig. 3). 

In the final remodeling phase, the temporary granulation matrix is replaced with organized type I collagen, and myofibroblasts contract the wound before undergoing apoptosis. MMPs degrade excess ECM components, while TIMPs ensure controlled remodeling (Rodrigues et al., 2019[120]; Xue and Jackson, 2015[154]). Peptides like Thymosin β4 plays a critical role in scar modulation by reducing myofibroblast persistence, limiting TGF-β-driven fibrosis, and regulating MMP activity, ultimately leading to faster wound healing as shown in Figure 3e(Fig. 3).

### Wound healing peptides, structure, classification, and functional diversity

Peptides are organic molecules consisting of chains of amino acids (ranging from about 2 to more than 50) linked by peptide bond. Peptides exhibit diverse pharmacological effects, including antimicrobial, antihypertensive, anti-inflammatory, and anticancer effects (Zhou et al., 2023[170]). A polypeptide is a longer, continuous, unbranched peptide chain, When its molecular mass reaches 10,000 Da or more, it is referred to as a protein (Feng et al., 2022[35]). Chains of 4 - 20 amino acids are termed oligopeptides, and include dipeptides, tripeptides, and tetrapeptides (Nasadyuk, 2021[97]). Peptides can be broadly classified according to their origin, chain length, biological role, and structural properties. Currently, peptides have attracted significant attention due to their ability to modulate diverse biological pathways and enhance cellular and molecular interactions (Gori et al., 2023[45]). 

A notable example is the Arg-Gly-Asp (RGD) peptide sequence, which enhances fibroblast recruitment and migration, thereby accelerating the inflammatory and proliferative phases of wound healing (Chen et al., 2025[19]). The following sections further explain different classes of peptides with wound healing properties.

### Wound healing peptides from micro-organisms

Antimicrobial peptides (AMPs) are a diverse group of naturally occurring molecules found in a wide range of organisms, including humans, toad, and mice. They are generally low in molecular weight, cationic, and amphipathic. Their amphipathic nature allow them to insert into the lipid bilayers and disrupt the microbial cell membranes (Feng et al., 2022[35]; Travkova et al., 2017[141]). AMPs typically act through mechanisms such as pore formation, membrane disintegration, and intracellular targeting (inhibition of protein or DNA synthesis), thereby providing broad-spectrum activity against bacteria, fungi, viruses, and even cancer cells (Le et al., 2017[76]). 

AMPs contribute significantly to the wound-healing process through their antimicrobial, immunomodulatory, and regenerative properties (Nasseri and Sharifi 2022[98]). Their immunomodulatory functions include regulating cytokine production, influencing immune cell recruitment to the wound site, suppressing excessive inflammation by downregulating TNF-α, IL-6, and other pro-inflammatory mediator, and enhancing phagocytosis by modulating macrophage activity to facilitate the clearance of necrotic tissue and pathogens (Duan et al., 2022[29]), In addition, AMPs are highly effective against multidrug-resistant bacteria, as they target bacterial membranes, making it difficult for pathogens to develop resistance (Pervin and Hassan, 2021[105]). 

Many AMPs adopt α-helical or β-sheet conformations that enhance their stability and interaction with bacterial membranes. Also, their relatively short peptide chains enable rapid diffusion through tissue, making them particularly effective for deep wound penetration (Zhang et al., 2025[164]). A notable example of an AMPs is Nisin, produced by *Lactococcus lactis*. It binds to lipid II on bacterial membranes, forming pores that lead to ion leakage and bacterial cell death. Its potent antimicrobial effect primarily targets Gram-positive pathogens, thereby minimizing wound infections and supporting tissue regeneration (Khan et al., 2023[68]). Other types of AMPs are summarized in Table 2(Tab. 2) (References in Table 2: Ahmad et al., 2024[5]; Araujo et al., 2022[9]; Cao et al., 2018[14]; David et al., 2016[23]; Di Grazia et al., 2015[27]; Dyrda-Terniuk and Pomastowski, 2023[30]; Fensterseifer et al., 2015[36]; Guryanova and Ovchinnikova, 2022[49]; Hoq et al., 2011[55]; Huang et al., 2017[58]; Khan, 2022[68]; Kim et al., 2015[72]; Kurek-Górecka et al., 2021[75]; Li et al., 2024[78]; Misra et al., 2024[91]; Nasseri and Sharifi, 2022[98]; Ong et al., 2020[103]; Shahzad, 2015[124]; Shini et al., 2022[126]; Song et al., 2019[131]; Takahashi et al., 2021[138]; Wang et al., 2018[143]; Wang et al., 2023[145]; Yacoub et al., 2020[155]; Yan et al., 2020[158]; Yang et al., 2024[159]; Yu et al., 2025[162]; Zhao et al., 2016[167]; Zhao et al., 2019[166]; Zhou et al., 2021[169]; Zhou et al., 2023[170]). 

### Wound healing peptides from animals

Animal-derived peptides have been shown to accelerate wound healing, prevent scar formation, and contribute to infection control at the wound site. As they are derived from natural animal proteins, these peptides are generally considered safe and reliable. They are sourced from a wide range of animals, including amphibians, insects, marine organisms, and mammals (Fan et al., 2024[33]).

Structurally, animal-derived peptides are typically composed of short amino acid sequences (ranging from 12-50 residues) that may be linear or cyclic and often adopt secondary structures such as α-helices and β-sheets. They are amphipathic, containing both cationic and hydrophobic domains, which enable them to interact with microbial membranes and as well as host cells, thereby exerting antimicrobial and wound-healing effects (Sun et al., 2024[137]; Ma et al., 2024[83]). For example, Bovine Lactoferrin (BLF) is an iron-binding glycoprotein derived from *bovine colostrum*, with each lobe capable of binding a single Fe³⁺ ion. Its structure enables it to inhibit microbial growth by sequestering iron, which bacteria need to survive, and by disrupting microbial membranes. In wound healing, BLF modulates inflammation by downregulating key cytokines such as TNF-α and IL-6, and promotes ECM remodeling through the regulation of MMPs. It exerts positive effects across all phases of wound healing, homeostasis, inflammatory, proliferative, re-epithelization and remodeling phase (Dyrda-Terniuk and Pomastowski, 2023[30]; Shini et al., 2022[126]). Other types of animal-derived peptides are summarized in Table 2(Tab. 2).

### Wound healing peptides from plants

Plant-derived peptides are typically short sequences of 2-20 amino acids with molecular weights below 3 kDa, and are generated through the enzymatic hydrolysis of plant proteins (Nicolas-Espinosa et al., 2022[100]). Their small size and structural composition often featuring cationic and hydrophobic residues enable them to interact with and penetrate biological membranes. This facilitates various therapeutic effects, including modulation of inflammation, reduction of oxidative stress, antimicrobial activity, stimulation of cell proliferation, and remodeling of ECM, all of which are crucial to the skin repair process (Nirmal et al., 2024[102]; Fan et al., 2022[32]). In a burn-injury rat model, oral administration of soybean-derived peptides downregulated NF-κB pathway activation and reduced neutrophil and macrophage infiltration at wound sites. Treated animals also exhibited enhanced angiogenesis, indicated by elevated CD31 expression, and accelerated healing, achieving 67 % wound closure compared with 41 % in control animals by the eighth week (Zhao et al., 2019[166]). Other types of plant derived peptides are summarized in Table 2(Tab. 2).

### Wound healing synthetic peptides

In addition to natural peptides, synthetic peptides have gained a considerable attention as bioactive agents for accelerating wound healing. Their small size, ease of synthesis, and high tunability make them versatile tools that can be tailored for specific biological functions. They can be rationally designed or derived from natural protein sequences to target specific pathways involved in wound repair (Guan et al., 2022[46]; Md Fadilah et al., 2024[88]).

A notable example is A7-1, a synthetic peptide derived from a 13-residue sequence in silk fibroin, identified for its adhesive properties and ability to support early wound regeneration. A study demonstrated that topical application of A7-1 in mice accelerated early angiogenesis and granulation tissue formation, potentially through stabilizing local growth factor gradients (Jung et al., 2024[66]). Additional engineered peptides and their mechanisms of action are summarized in Table 3(Tab. 3) (References in Table 3: Chung et al., 2017[21]; Dzierżyńska et al., 2023[31]; Gawande et al., 2014[40]; Gomes et al., 2017[44]; Jiang et al., 2023[64]; Jung et al., 2024[66]; Liu et al., 2024[82]; Mndlovu et al., 2023[92]; Nakagami et al., 2017[96]; Pfalzgraff et al., 2016[106]; Singh et al., 2025[128]). Furthermore, to provide a concise overview of the strength of evidence, Table 4(Tab. 4) (References in Table 4: Ansari et al., 2021[8]; Cappiello et al., 2019[16]; Carretero et al., 2008[17]; Di Grazia et al., 2015[27]; Dzierżyńska et al., 2023[31]; Guarnera et al., 2010[47]; He et al., 2019[50]; Heunis et al., 2013[52]; Hoq et al., 2011[55]; Hu et al., 2023[57]; Jaramillo et al., 2023[62]; John et al., 2023[65]; Karimi et al., 2021[67]; Kim et al., 2015[72]; Koohzad and Asoodeh, 2023[73]; Lebedeva et al., 2017[77]; Liu et al., 2014[80]; Liu et al., 2014[81]; Mahlapuu et al., 2021[84]; Mndlovu et al., 2023[92]; Mouritzen et al., 2019[95]; Nakagami et al., 2017[96]; Philp et al., 2004[107]; Pickart et al., 2015[108]; Radek et al., 2008[114]; Raheem et al., 2024[116]; Rivera-Sanchez et al., 2025[117]; Shini et al., 2022[126]; Simonetti et al., 2008[127]; Song et al., 2025[129]; Sun et al., 2019[136]; Tomioka et al., 2014[140]; Uşaklıoğlu and Çakan, 2023[142]; Wigger-Alberti et al., 2012[148]; Yaraguppi et al., 2023[160]; Zhang et al., 2020[165]; Zhao et al., 2019[166]; Zhou et al., 2021[169]) summarizes wound healing peptides for which results have been independently reproduced across different study types.

## Hydrogels for the Delivery of Therapeutic Peptides: Shielding, Synthesis, and Wound Targeting

Hydrogels are three-dimensional networks of hydrophilic polymers that can absorb large amounts of water while retaining a semi-solid structure. With water content often exceeding 90 %, hydrogels provide a moist environment that facilitates tissue repair. They can be tuned to incorporate bioactive components (e.g., antimicrobials, growth factors, or cells) that actively promote healing (Xiang et al., 2020[152]; Khan et al., 2016[69]). The swollen hydrogel matrix can also absorb excess wound exudate, helping to regulate tissue fluids while maintaining hydration. Importantly, hydrogel dressings provide a degree of mechanical protection, as they are conformable and cushion the wound against external stresses. Unlike traditional dry gauze, hydrogels do not adhere tightly to the granulating wound bed, thereby minimizing pain and tissue damage during dressings (Xiang et al., 2020[152]; Ahmed, 2015[7]; Feng et al., 2024[34]). These characteristics, moisture retention, non-adherence, and softness, make hydrogels particularly suitable for dry or painful wounds, including burns, necrotic wounds, and chronic ulcers.

Beyond passive protection, hydrogels act as an active scaffold for tissue regeneration. Their porous, hydrated structure mimics the natural ECM, allowing cells to infiltrate and proliferate within the wound site (Radulescu et al., 2022[115]). In particular, peptide-based hydrogels rely on peptides that self-assemble into nanofibrous networks through non-covalent interactions such as β-sheet hydrogen bonding and hydrophobic forces (Dou and Feng, 2017[28]). A hallmark of peptide hydrogels is their biomimicry of the ECM, they form nano-scale fibrous scaffolds resembling collagen fibrils and proteoglycan networks, creating a cell-friendly matrix that supports cell adhesion and migration (Wang et al., 2020[146]). Peptides can be designed to include specific biochemical signals (e.g., the RGD cell-adhesion motif or growth factor-binding sequences), endowing these hydrogels intrinsic bioactivity not easily achieved in conventional synthetic polymer gels (Mndlovu et al., 2023[92]). Peptide hydrogels are generally biocompatible and biodegradable *via* proteolytic enzymes because they are composed of natural and short chains of amino acid, thereby minimizing chronic foreign-body reactions (Wu et al., 2022[151]).

**Figure 4:**
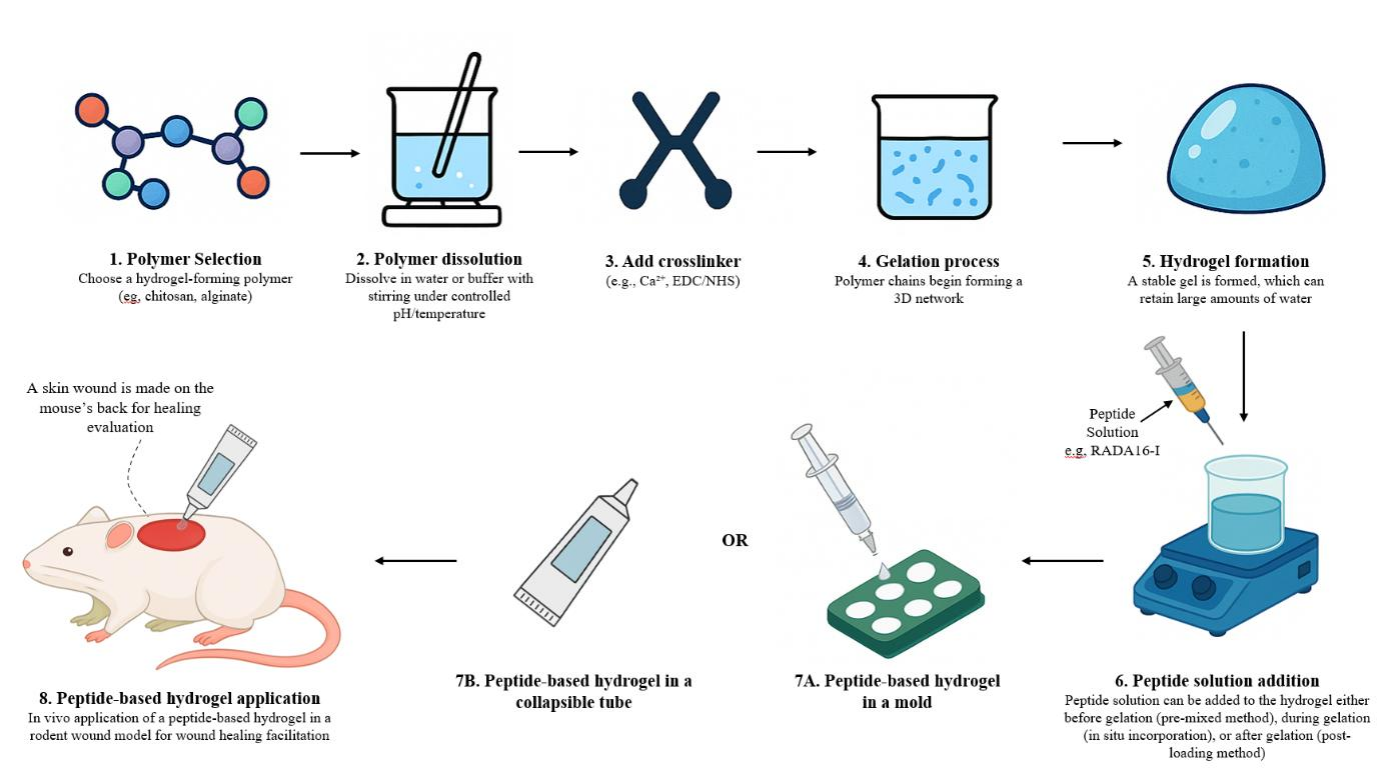
A schematic illustration that shows the preparation and application of peptide-based hydrogels for wound healing.

The formulation of hydrogels incorporated with peptide typically begins with the selection or synthesis of short peptide sequences capable of self-assembly. These peptides are then dissolved in an appropriate solvent to initiate the process. Crosslinking agents or environmental triggers are then used to induce self-assembly into a hydrogel matrix. Therapeutic peptides such as RADA16-I may be incorporated to boost biological activity. The hydrogel is subsequently molded, sterilized, and applied to wound models (e.g., mice) for *in vivo* evaluation (Dzierżyńska et al., 2023[31]; Stefanov et al., 2017[135]) as illustrated in Figure 4(Fig. 4). 

### Approved and commercially available peptide-loaded hydrogels in wound care

Peptide-based hydrogels can enhance nearly all phases of wound healing. For example, during the homeostasis phase, they can aid by rapidly forming a physical barrier and promoting clot formation. In a rat bleeding model, h9e peptide solution achieved hemostasis 82 % faster than a commercial hemostatic agent (Celox™) by rapidly gelling and concentrating platelets and clotting factors at the bleeding site (Guan et al., 2022[46]; Carter et al., 2021[18]). 

Additionally, peptide-based hydrogels can facilitate the inflammatory phase through their intrinsic antimicrobial and immunomodulatory functions (Kharaziha et al., 2021[70]). A notable example is a hydrogel functionalized with the thrombin-derived peptide TCP-25, designed to address bacterial infection and inflammation. TCP-25 not only exhibits broad-spectrum antimicrobial activity but also binds bacterial endotoxins (e.g., lipopolysaccharide) neutralizing their inflammatory effects. In infected wound models, a TCP-25 peptide hydrogel eradicated *Staphylococcus aureus* and *Pseudomonas aeruginosa* while concurrently reducing pro-inflammatory cytokine levels in the local tissue (Dahlman et al., 2021[22]). 

Moreover, peptide hydrogels can significantly enhance the proliferative phase by serving as a bioactive scaffold for cell growth (Im et al., 2018[60]). Because peptide matrices closely resemble native ECM, they promote keratinocyte and fibroblast migration into the wound and support their proliferation (Xiang et al., 2020[152]). For example, in a study using a chitosan-based film-forming gel containing tyrothricin, researchers observed accelerated healing in rat wound models, including burns, abrasions, and excisions. Treated wounds showed enhanced granulation tissue formation and epithelialization compared with control groups. Additionally, histological analyses revealed increased fibroblast proliferation and angiogenesis, leading to a faster and smoother transition to tissue regeneration (Kim et al., 2015[72]).

In the final remodeling phase, peptide hydrogels can influence remodeling and improve healing in earlier phases, eventually reducing scar formation (Song et al., 2020[130]). For instance, a study investigating a resveratrol-loaded self-assembling peptide hydrogel, RADA-PDGF2, in a rat model demonstrated its beneficial effects on wound healing. Treated wounds displayed more organized collagen deposition and nearly normal epidermal architecture at the completion of healing, with minimal scar tissue. Furthermore, histological analyses showed that collagen fibers in treated wounds were aligned similarly to those in uninjured skin, in contrast to the disorganized bundles typically observed in scar tissue (Deptuła et al., 2023[26]). Thus, peptide-based hydrogels not only accelerate the early stages of healing but also improve the overall quality of repair. Table 5(Tab. 5) (References in Table 5: Abbasi Aval et al., 2022[2]; Capella-Monsonís et al., 2020[15]; Gil et al., 2022[42]; Guiotto et al., 2024[48]; Michaels et al., 2024[89]; Mohapatra et al., 2021[93]) lists peptide-based hydrogels that are available in the current market.

### Recent advancements in peptide-based hydrogels

Recent research has achieved remarkable advances in the functionalization and performance of peptide hydrogels for wound healing. For example, in 2023, Huang et al., engineered an “all-peptide” hydrogel using modified γ-polyglutamic acid and RGD-containing peptides, which could be 3D-printed with living cells. Endothelial cells overexpressing VEGF were printed within this peptide matrix, creating a living scaffold that continuously released VEGF, promoted angiogenesis. In diabetic wound models, this VEGF-eluting peptide hydrogel significantly accelerated wound closure and enhanced tissue regeneration by reducing inflammation and hypoxia (Huang et al., 2023[59]). 

In 2025, Chen et al. reported a self-healing hydrogel composed of food-derived peptides that simultaneously addresses infection, inflammation, and hemostasis. The hydrogel was formed by cross-linking egg-white proteins with oxidized polysaccharides and incorporating a copper-bound tripeptide (GHK-Cu) known for its regenerative activity. The resulting gel demonstrated broad-spectrum antibacterial and anti-inflammatory effects and rapidly stopped bleeding by adhering to tissues (Filipczak et al., 2021[37]). In infected wound models, the GHK-Cu peptide hydrogel significantly accelerated healing, achieving ~95 % wound closure by day 12 compared with 65 % in controls, primarily by promoting neovascularization and tissue regeneration (Chen et al., 2025[20]). 

Another advancement is the development of dual-action anti-infective peptide hydrogels. Puthia et al. (2020[110]) created a hydrogel incorporating TCP-25, a peptide derived from thrombin that not only kills bacteria but also neutralizes inflammatory endotoxins (PAMPs). In mouse and pig wound models, a TCP-25 hydrogel eradicated *MRSA* and *Pseudomonas aeruginosa* infections and significantly reduced pro-inflammatory cytokines in the wound environment, thereby expediting healing (Puthia et al., 2020[110]). This study highlights a strategy of functionalizing a polymer hydrogel with a host-defense peptide to create a dual-action dressing capable of reducing bacterial biofilm and excessive inflammation.

Peptide hydrogels are also being designed to respond to wound microenvironmental cues. For example, pH-switchable peptide assemblies have been developed to release therapeutics in infected wounds (Li et al., 2022[79]). Many peptide hydrogels are injectable or sprayable, leveraging their shear-thinning behavior. A notable property of certain peptide formulations (e.g., the UD-developed peptides in G4Derm) is their ability to be applied as a liquid and rapidly gel *in situ* upon contact with tissues, conforming to irregular wound shapes (Roberts, 2024[119]). This shear-thinning and rapid recovery behavior is particularly advantageous for filling deep or tunneling wounds where pre-formed dressings cannot reach. Such advancements greatly enhance the applicability of peptide hydrogels in complex wound scenarios. Table 6(Tab. 6) (References in Table 6: De Leon-Oliva et al., 2023[24]; Foley and Lau, 2016[38]; Hosoyama et al., 2019[56]; Mandla et al., 2019[86]; Puthia et al., 2020[110]; Robert et al., 2022[118]; Roberts, 2024[119]; Seow et al., 2016[123]; Wei et al., 2019[147]; Xiao et al., 2016[153]; Yadav et al., 2022[156]; Zhang et al., 2024[163]; Zhou et al., 2022[168]) summarizes additional recent progress in peptide hydrogel research. 

Additionally, a range of peptide-containing hydrogels is currently under investigation for skin regeneration. For example, Granexin^®^ Gel (containing αCT1) has advanced to Phase III trial in diabetic foot ulcers, while SLI-F06, a fibromodulin-mimetic peptide hydrogel, has completed Phase I/IIa testing in post-surgical scars (Freedman et al., 2023[39]). The emerging clinical pipeline of peptide hydrogels ranging from anti-scarring agents to antimicrobial and angiogenic dressings reflects a rapidly maturing field poised to deliver novel bioactive wound therapies. Examples of hydrogels currently in clinical trials but not yet approved by regulatory agencies are presented in Table 7(Tab. 7) (References in Table 7: Attik et al., 2023[10]; Banerjee et al., 2025[11]; Deliencourt- Godefroy et al., 2025[25]; Freedman et al., 2023[39]; Gelain et al., 2021[41]; Niemeyer et al., 2022[101]; Yaguchi et al., 2021[157]; Ye et al., 2022[161]).

## Future Directions of Peptides in Wound Healing

An innovative development in this field is the use of wound-healing peptides that selectively bind to injured tissues. One example is the cyclic peptide CAR (CARSKNKDC), which targets angiogenic vasculature and accelerates wound closure and re-epithelialization by enhancing keratinocyte migration *via* syndecan-4 signaling (Maldonado et al., 2023[85]). Such approaches enable targeted modulation of healing pathways, making them particularly suitable for chronic or non-healing wounds.

Another promising innovation as suggested by Huang et al. is the use of 3D bioprinting to create customized peptide-based scaffolds. Where endothelial cells overexpressing VEGF were bio-printed into a peptide hydrogel scaffold, creating a bioactive matrix that substantially enhanced vascularization and wound closure in diabetic models. This demonstrates the potential of integrating peptide science with additive manufacturing to produce personalized, therapeutic wound grafts (Huang et al., 2023[59]).

Moreover, peptides can also be incorporated into smart dressings that can monitor and respond to the wound environment. For example, wearable biosensors functionalized with peptides can be developed to detect infection-related biomarkers like *S. aureus* enzymes and inflammation markers such as MMP-13. These dressings can wirelessly transmit diagnostic data, enabling real-time monitoring and early intervention. 

## Conclusion

Peptides have rapidly emerged as an effective therapy for advanced wound care due to their ability to modulate the complex biological processes that underlie tissue repair. Unlike traditional topical therapies that often target single aspects of healing, peptides offer a multifunctional approach by acting as antibacterial agents, angiogenic stimulants, immune-modulators, and regenerative inducers. Their capacity to interact with cellular receptors and modify intracellular signaling pathways across all stages of wound healing makes them an ideal candidate for managing chronic wounds.

Therapeutic peptides, owing to their short amino acid chains, can be tailored or naturally sourced to target specific molecular pathways and regulate key mediators like VEGF, MMPs, and TGF-β in wound healing. They offer favorable pharmacokinetics including high tissue permeability, low immunogenicity, and predictable degradation, but face challenges of enzymatic breakdown and rapid clearance. To overcome these, advanced delivery systems such as hydrogel encapsulation are employed to enhance stability, efficacy, retention, and controlled release.

The future of peptide therapeutics in wound care lies in innovations at the intersection of biology, chemistry, and material science. Stimuli-responsive peptides, capable of releasing therapeutic cues in response to pH, enzymatic activity, or oxidative stress, are currently under active investigation. Integration with biosensors, wearable electronics, and 3D bioprinting technologies may enable real-time diagnostics and personalized therapeutic delivery. Furthermore, the exploration of ultrashort peptides, cyclopeptides, and peptide-mimetic analogs presents new opportunities to overcome the traditional limitations of peptide-based therapeutics.

## Notes

Shaik Nyamathulla and Syed Mahmood (Department of Pharmaceutical Technology, Faculty of Pharmacy, Universiti Malaya, 50603 Kuala Lumpur, Malaysia; Phone number: +6018-267-9732; E-mail: syedmahmood@um.edu.my) contributed equally as corresponding author.

## Declaration

### Conflict of interest

The authors declare that there are no commercial or financial relationships that could be construed as a potential conflict of interest in the preparation and publication of this review.

### Artificial Intelligence (AI) - Assisted Technology

No AI tool was used for the writeup of this manuscript.

### Author contributions

Rafl M. Kamil: Conceptualization, Methodology, Data curation, Formal analysis, Writing original draft. Shaik Nyamathulla: Conceptualization, Funding acquisition, Resources, Supervision, Review and editing. Syed Mahmood: Conceptualization, Resources, Supervision, Review and editing.

### Acknowledgments

The authors would like to express their utmost gratitude and appreciation to Universiti Malaya for funding a research project (Universiti Malaya Research Excellence grant -UMREG071-2024) related to the peptide-based hydrogels for wound healing. Further, the work on peptides is supported by the Ministry of Higher Education, Malaysia by the Fundamental Research Grant Scheme (FRGS) grant (FP-048-2020) to investigate the role of peptides in chronic wounds.

## Figures and Tables

**Table 1:**
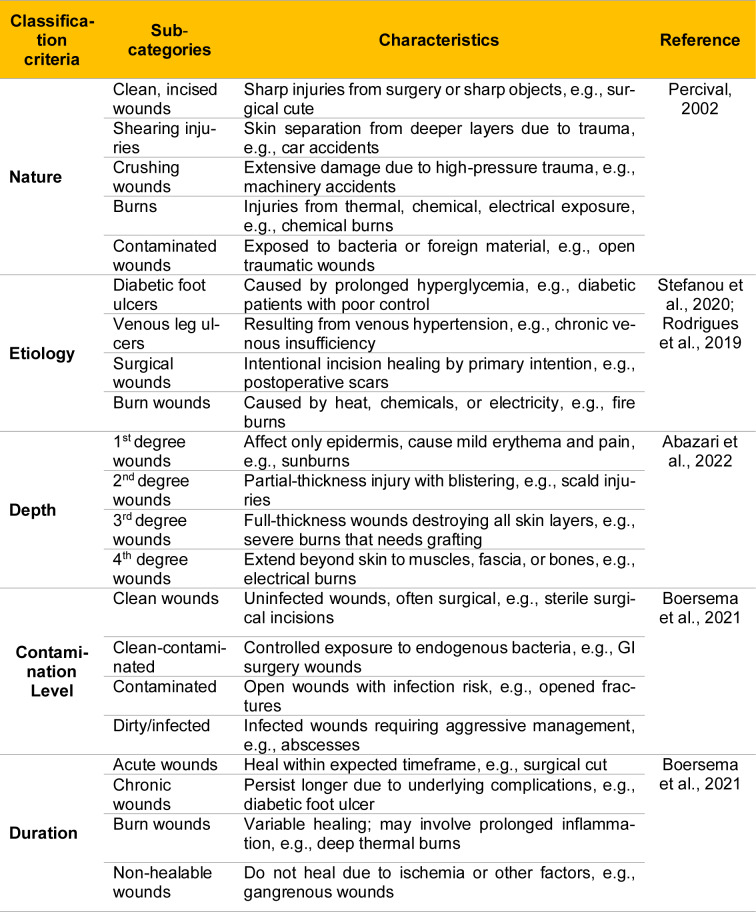
Classification of wounds based on different wound characteristics

**Table 2:**
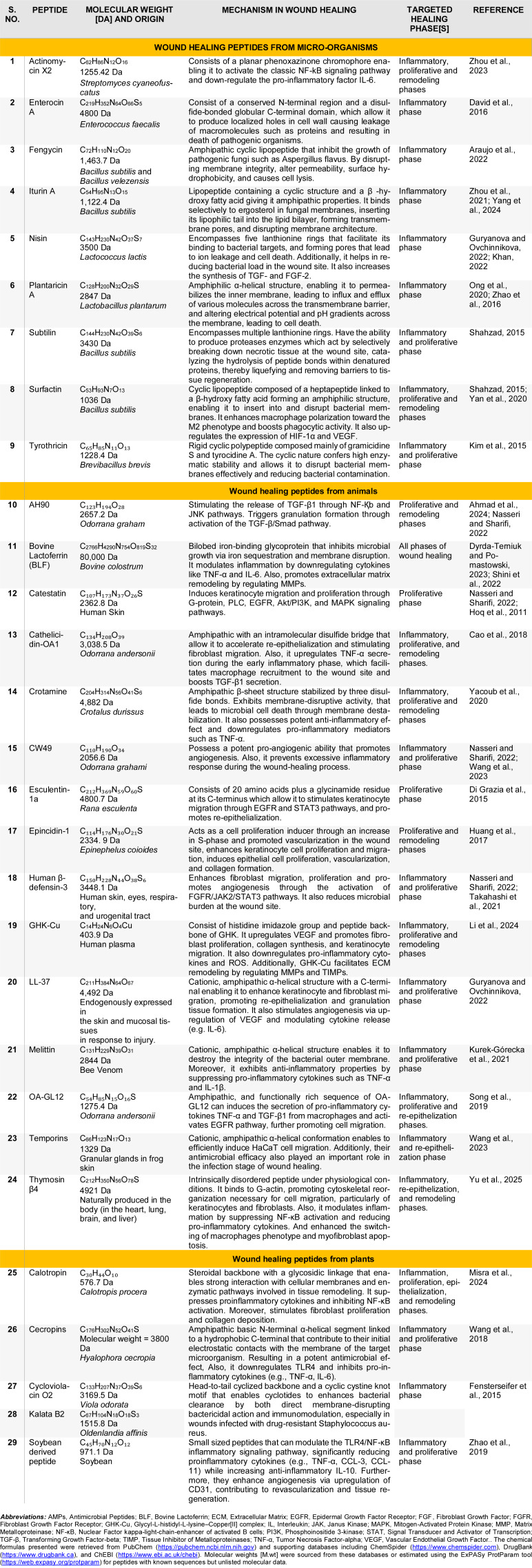
Bioactive peptides derived from microbial, animal, and plant sources with documented roles in wound healing

**Table 3:**
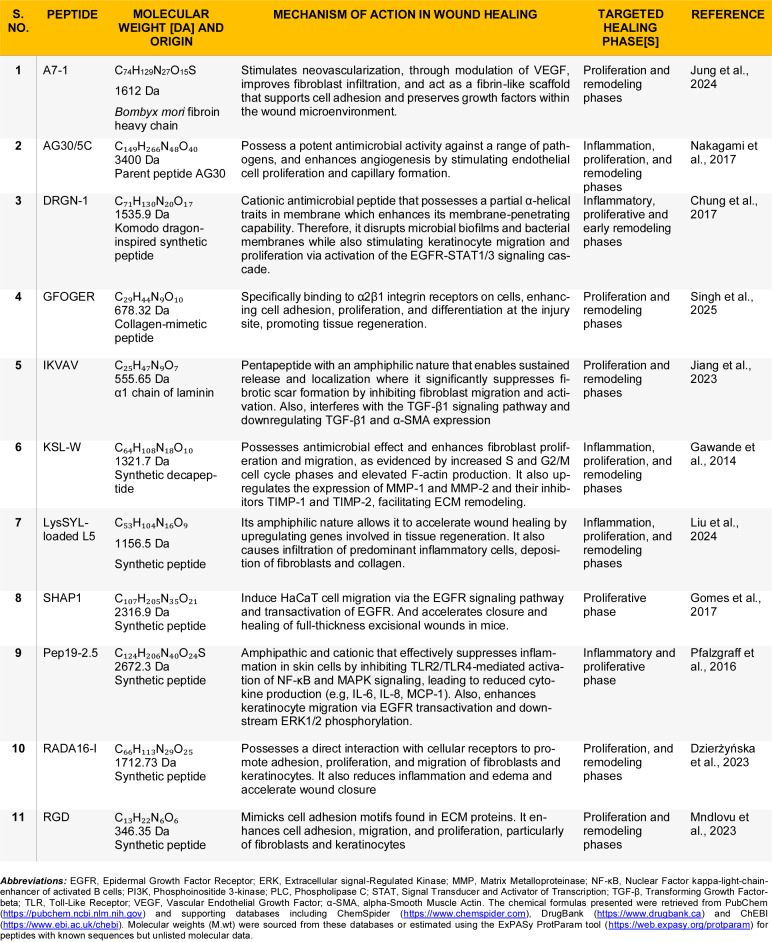
Synthetic peptides engineered for wound healing applications

**Table 4:**
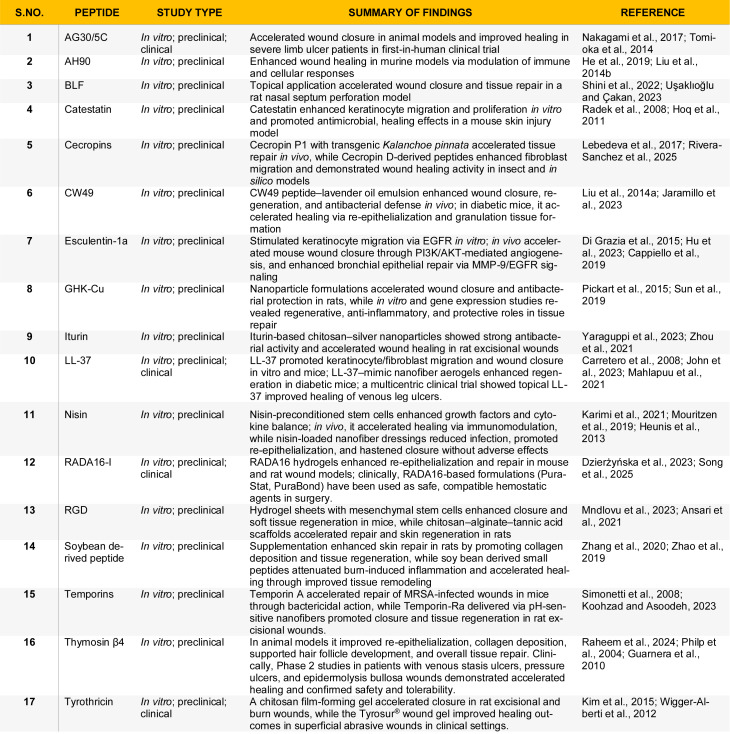
Wound-healing peptides with details of the studies replicated by independent research groups

**Table 5:**
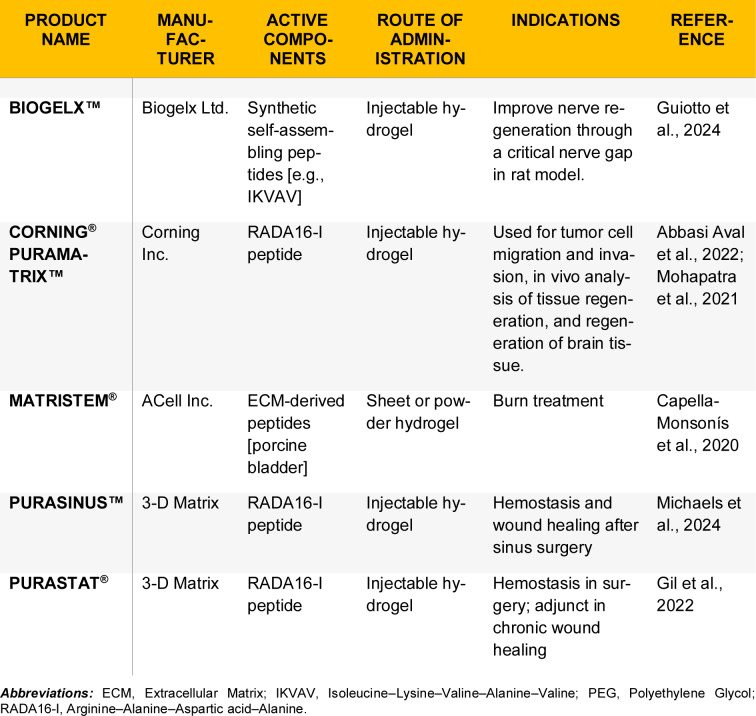
List of commercial peptide-based hydrogels in the current market

**Table 6:**
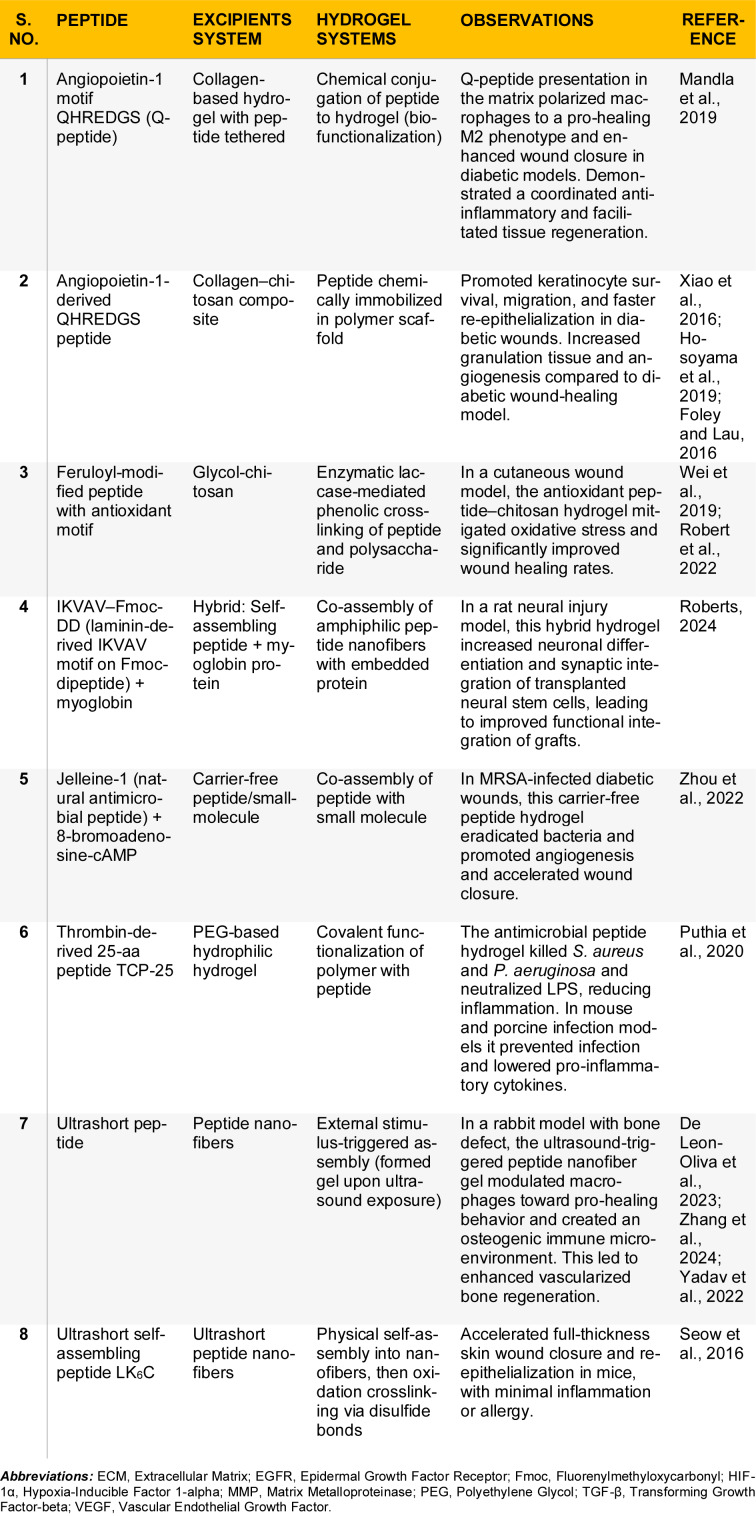
Recent advances on peptides as tissue regenerative wound healing agents for skin conditions

**Table 7:**
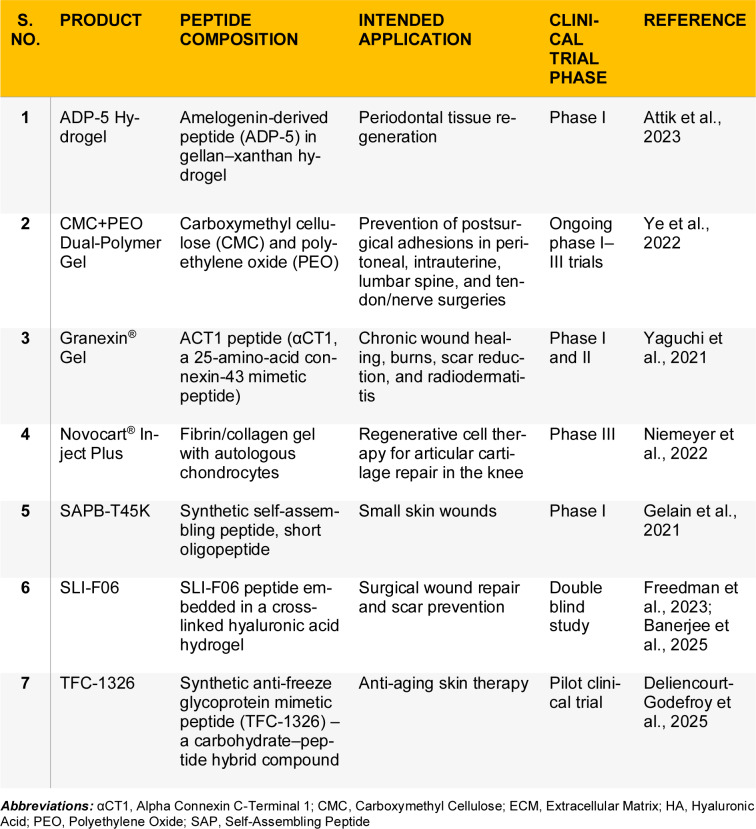
List of peptide hydrogels that are currently under investigation in clinical trials

**Figure 1:**
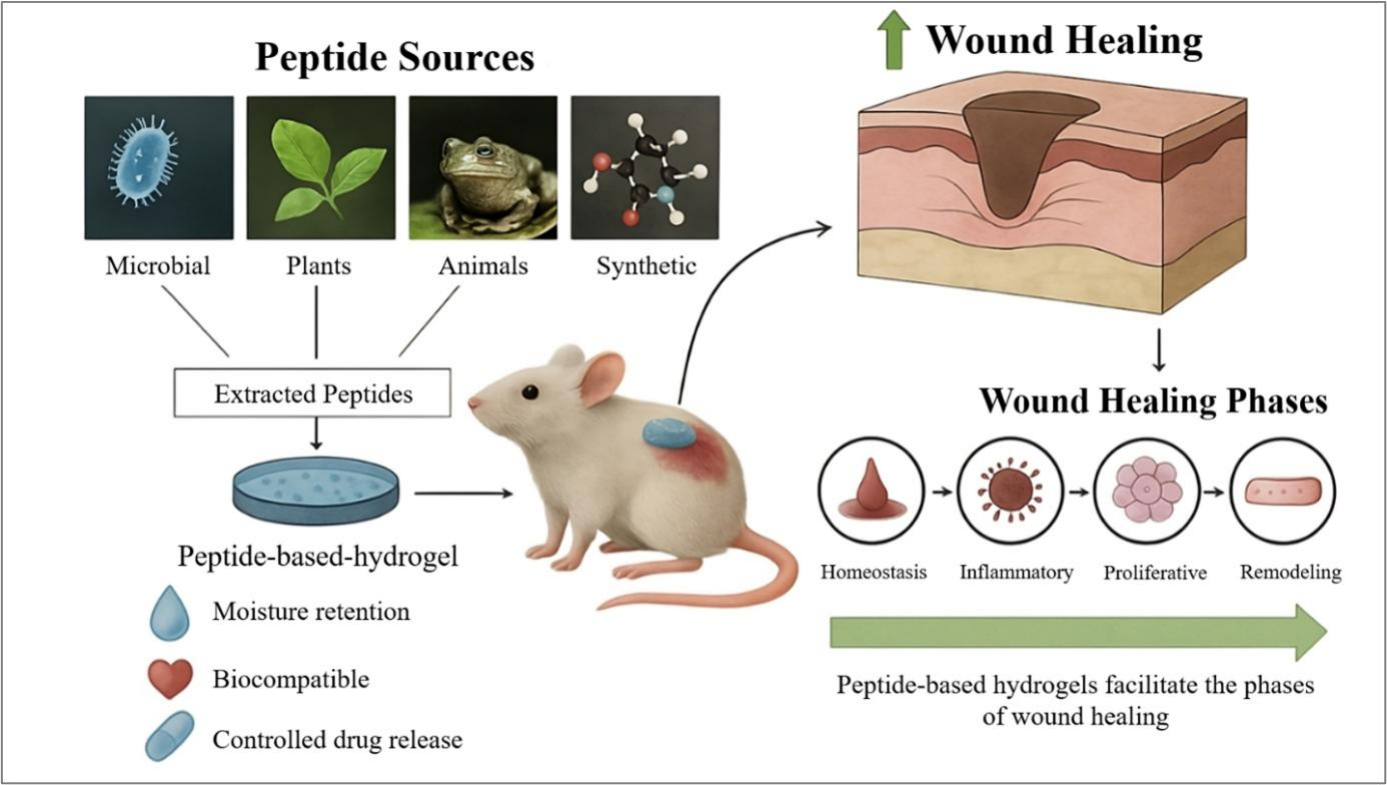
Graphical abstract
